# Financial Support or Emotional Companion: Childbearing Motivations on Children’s Development in China

**DOI:** 10.3389/fpsyg.2021.690980

**Published:** 2021-06-04

**Authors:** Xiaodong Jiang, Huaxue Cui, Tianfeng Shi

**Affiliations:** ^1^College of Business, Shanghai University of Finance and Economics, Shanghai, China; ^2^Faculty of Management, McGill University, Montreal, QC, Canada

**Keywords:** childbearing motivations, financial support, emotional companion, well-being, academic performance, self-esteem

## Abstract

A preference for having a son has existed among Chinese parents for centuries due to, in part, sons having to provide financial support to elderly parents, while married daughters do not have this responsibility under Confucianism. Thus, this study examined the influence of parents’ childbearing motivation (financial support or emotional companion) on children’s development (academic performance and well-being) utilizing empirical data from the 2012 China Family Panel Studies. This study included 1,541 children (aged 10–15 years) and their parents who were surveyed *via* a questionnaire. Using exploratory factor analysis, two dimensions of parents’ childbearing motivation were identified namely, utilitarian and psychological motivation. Furthermore, the invariance of the measurement model across the female and male group was tested. Then, results from structural equation modeling showed that parents’ childbearing motivation, particularly expected utilitarian benefits, decreased children’s expectation of the highest education, thus, worsening children’s academic performance. Alternatively, emotional/psychological motivation appeared to increase children’s self-esteem, thus, improving children’s well-being. Furthermore, gender differences were also observed. These findings have provided important insights into how childbearing motivations influence children’s development, thus, can be utilized to ensure positive development of future children in China.

## Introduction

Childbearing is an important issue as, from the perspective of human society, it is related to the preservation of the human species. From a family perspective, it is related to the survival of the family line, especially under familial cultural traditions. From an individual perspective, childbearing is related to human well-being because children are the foundation of people’s greatest joys and the source of their greatest sorrows (Nelson et al., 2014). Due to these different perspectives, people may or may not plan to raise a child. However, various motivations behind the decision to become a parent exist.

As the fertility rate is crucial to a country’s development, abundant research has explored various factors influencing people’s childbearing intentions including tempo (i.e., the timing of childbirth) and quantum (i.e., the total number of children; Miller, 1995; Bongaarts and Feeney, 1998; Kohler et al., 2002; Miller et al., 2004; Billingsley, 2010; Iacovou and Tavares, 2011; Bein et al., 2020). The relationships between childbearing intention and parents’ well-being, as well as children’s development, have also been widely debated (Crissey, 2005; Kohler et al., 2005). For example, an unwanted pregnancy may reduce maternal behavior after controlling for the family background, which adversely affects infant and child health (Joyce et al., 2000).

Previous literature on people’s childbearing behavior mainly focuses on the antecedents influencing people’s willingness to bear a child. For example, psychological traits (e.g., subjective norms and perceived behavioral control) and developmental experiences (e.g., education, work, religion, and parental relationships) could predict people’s childbearing intention (Billari et al., 2009; Miller et al., 2011; Balbo et al., 2013). In addition, health (exposure to sexually transmitted infections and HIV) and social determinants (poor prospects for future education and economic security) may have negative childbearing intentions for younger parents (Kamila et al., 2018). However, to have a child is among individuals’ most important and meaningful decisions, with far-reaching implications. Despite evidence linking this decision to a wide variety of consequences, little is known about what motivates people to have children, and even less about the long-term effects of different childbearing motivations on parenting and child adjustment (Nachoum et al., 2021).

Although some dimensions of childbearing motivation have been identified (Nauck, 2007; Guedes et al., 2015), limited research has investigated how parents’ childbearing motivations influence children’s performance or well-being. Nachoum et al. (2021) shows that prenatal maternal autonomy and controlled childbearing motivations are related to child behavior problems through parenting styles. Moreover, some of the childbearing related topics focus on delayed parenthood (Beets et al., 2011; Mills et al., 2011; Van de Kaa, 2011) and how childbearing motivations may affect the fertility preferences in reproductive-age women; however, do not appear to have any influence on the actual number of children they have (Irani and Khadivzadeh, 2018).

However, why people want to bear children or where the value of children resides for parents has been less focused on. Hoffman and Hoffman (1973) identified nine types of childbearing motivations but did not give a clear classification of these. Later research revealed additional systematic dimensions of childbearing motivations (Nauck, 2001; Nauck and Klaus, 2007; Guedes et al., 2015). Although these different motivations have been identified, little research has explored the relationship between parents’ motivations and children’s development. Thus, this study is a direct response to this gap in literature and seeks to examine the effect of parents’ different childbearing motivations on children’ development and the underlying mechanisms. To this end, this study attempts to explain a common phenomenon in China namely, whether holding a realistic goal when raising a child (obtain financial support) or not (enjoy emotional companion) will affect children’s development, including children’s academic performance and well-being.

## Theory and Hypothesis Development

Previous literature investigated the relationship between intended or unintended childbearing and children’s development (Joyce et al., 2000; Korenman et al., 2002). If children were born in the unintended condition, they were more likely to have worse health and development assessments (Crissey, 2005). However, few studies considered whether intended childbearing with different motivations may lead to different developmental outcomes. Nauck et al. (Nauck, 2001; Nauck and Klaus, 2007) emphasize two key dimensions: economic or utilitarian reasons (e.g., the economic contribution of children to the well-being of the household, their contribution to household chores, and their role in providing care to elderly parents) and psychological or emotional reasons (e.g., the reinforcement of emotional ties and expressive stimuli following an interaction with children). Guedes et al. (2015) proposed two additional dimensions: social or normative reasons (e.g., social or familial norms and pressure and religious or moral mandatories) and biological or physical reasons (e.g., maternal or paternal appeal and pressure of the biological clock). These motivations are regarded as positive because they focus on the benefits of raising a child without distinguishing between external or internal benefits. In addition, several negative childbearing motivations were also identified such as (1) discomforts of pregnancy and childbirth, (2) fears and worries of parenthood, (3) negatives of childcare, and (4) parental stress (Miller, 1995). Thus, negative childbearing motivations may negatively impact children’s development; however, whether positive childbearing motivations are beneficial to children’s development is yet to be determined.

In addressing how parents’ positive childbearing motivations influence children’s development differently, this study draws upon self-determination theory (SDT; Deci and Ryan, 2000). SDT postulates three innate psychological needs that are essential for human development, that is, the need for competence (i.e., feeling effective), autonomy (i.e., experiencing a sense of volition and psychological freedom), and relatedness (i.e., feeling loved and cared for). Ryan and Deci (2000) indicated that humans have an inherent tendency to seek out novelty and challenges, extend and exercise their capacities, explore, and learn; however, social and environmental factors, which reduce the feelings of autonomy and relatedness, can undermine this tendency and discourage them from being realized. Parents’ childbearing motivation is an often-neglected factor that may facilitate or undermine children’s development through reducing children’s feelings of autonomy and relatedness. To illustrate, if parents raise a child to recognize realistic goals (e.g., obtain financial support when they retire), children will be encouraged to accomplish this goal, intentionally or unintentionally, by their parents. Therefore, these children cannot experience psychological freedom and choice. Contrastingly, maternal autonomy support predicts more exploratory behavior in infants (Frodi et al., 1985). Thus, children lacking a feeling of autonomy are less motivated to explore new things and may have worse development. However, if parents raise a child motivated by enjoyment of the process, parents will devote more time to the child; thus, the child will feel more related to their parents. Anderson et al. (1976) found that children’s motivation to work on an interesting task were reduced if an adult was present and ignored them without responding to their initiations. Thus, a lack of a feeling of relatedness hinders children’s development. In this study, two aspects of children’s development (academic performance and well-being) were considered. Therefore, the following hypothesis is proposed:

*Hypothesis 1:* Different childbearing motivations have different (even opposite) effects on children’s academic performance and well-being.

Previous literature identified a famous concept of the “value of children” (VOC), which refers to the function children serve or the needs they fulfill for parents and is a commonly used concept to explain childbearing motivations (Hoffman and Hoffman, 1973; Friedman et al., 1994; Nauck, 2007). In turn, how people view the value of children can be interpreted as their childbearing motivation. A frequently cited notion states that people’s self-assessments are significantly shaped by the feedback they receive from others (Shrauger and Lund, 1975), especially feedback from significant others (e.g., parents; Gecas, 1971). The VOC is considered as parents’ prior evaluation of children, which will explicitly or implicitly influence children’s self-assessments. Self-assessment, more commonly referred to as self-esteem, refers to an individual’s overall feeling about their self. Abundant literature has shown a positive relationship between self-esteem and well-being (Valkenburg et al., 2006; Usborne and Taylor, 2010; Neff, 2011). For example, the effect of self-assessment on academic performance could be reflected by the child’s expectation of the highest education attainment. That is, if children have a higher self-assessment, they may have a higher education goal; thus, may devote more effort to their studies. Therefore, the following hypotheses are proposed:

*Hypothesis 2:* The effect of different childbearing motivations on children’s well-being is mediated by children’s self-esteem.

*Hypothesis 3:* The effect of different childbearing motivations on children’s academic performance is mediated by children’s expectation of the highest education.

In addition, a gender difference in terms of parents’ attitudes toward children exists in China, whereby a preference for sons has existed among Chinese parents for centuries (Hannum et al., 2009; Lee, 2012). The famous ideology in China, Confucianism, claims that women are inferior to men and play a limited role in households. However, after the one-child policy was implemented in China in 1979, female children’s access to education has increased as most families can afford the education cost for one child. Nonetheless, deeply affected by Confucianism, people still hold different expectations and childbearing motivations for male and female children. Therefore, the effect of childbearing motivation on children’s performance may differ across gender. As such, the following hypothesis is proposed:

*Hypothesis 4:* The effect of different childbearing motivations on children’s well-being is moderated by children’s gender.

## Materials and Methods

### Design and Participants

#### Participants and Data Collection

To test the hypotheses, the present study used publicly available data from the 2012 China Family Panel Studies (CEPS). The CFPS is a national social survey project implemented by the Chinese Social Science Survey Center of Peking University and targets households in all 25 provinces (municipalities and autonomous regions with approximately 95% of the population of China mainland) and reflects the changes in Chinese society, population, education, and health. The CFPS adopted a multistage, implicit stratification sampling method proportional to the size of the population. In 2012, the CFPS successfully interviewed 42,970 individuals in 12,725 households. The CFPS focuses on many research topics including economic activities, educational achievements, family relations, and health; thus, aligned with this study’s goals.

All family members including children (under 16 years of age) and adults were asked to complete their own personal questionnaires by themselves or with the help of others. Out of all the children included in the 2012 CFPS, children aged above 10 years were chosen as this age group had comparable academic performance. A total of 1,541 children aged 10–15 years in the 2012 CFPS was evident. Table 1 describes the basic demographics of the study sample. Among these children, 52.62% are boys, and 47.38% are girls. The percentage of children who come from urban areas is 37.52%, and the remaining 62.48% children are from rural areas. As these children were from 25 different provinces in China, the present study’s findings may be generalizable among Chinese children.

**Table 1:** Basic demographics of the study sample.

Variables	Mean or %	Std. dev.	Min	Max
Male	52.62			
Age	12	1.71	10	15
Urban	37.52			
***Province***				
Beijing	0.26			
Tianjing	0.39			
Hebei	5.26			
Shanxi	3.76			
Liaoning	6.04			
Jilin	1.04			
Heilongjiang	2.01			
Shanghai	2.40			
Jiangsu	1.82			
Zhejiang	1.04			
Anhui	1.43			
Fujian	1.10			
Jiangxi	2.99			
Shandong	3.70			
Henan	13.11			
Hubei	1.30			
Hunan	3.44			
Guangdong	11.94			
Guangxi	2.86			
Chongqing	1.10			
Sichuan	4.35			
Guizhou	7.33			
Yunnan	4.41			
Shaanxi	2.14			
Gansu	14.80			

### Analytical Model and Plan

**Figure 1:**
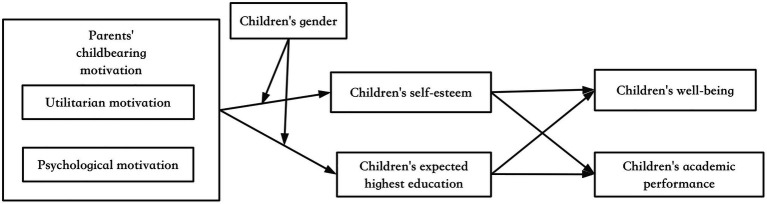
An analytical model with children’s psychological factors as mediators in Study 2.

Figure 1 illustrates the relationships examined in this study. It was hypothesized that parents’ childbearing motivations may influence children’s academic performance and well-being, and children’s self-esteem and expectation of the highest education may mediate these effects. In addition, these effects may be moderated by children’s gender.

To consider potential confounders, children’s living environment (rural vs. urban areas) and family income were included in the analyses as covariates. This is because, compared to children in rural areas, children in urban areas may have more resources regarding education, entertainment, social networks, and so forth. Family income was measured using the family’s net income between 2011 and 2012. In the present context, the number of children in a family was important because a child with more siblings may receive less family resources, both economically and psychologically. However, children in this study sample were born when the one-child policy was implemented; thus, most children in this sample did not have siblings.

#### Data Analysis

To determine the dimensionality of childbearing motivation, an exploratory factor analysis (EFA) using MPlus Version 8 was utilized. Hereafter, confirmatory factor analysis (CFA) was conducted to test the obtained factor structure conjectures. As this study involved a multigroup (male vs. female children) analysis to compare effects of childbearing motivations across gender, an additional measurement model invariance test was conducted. Furthermore, structural equation modeling (SEM) was applied to test the analytical model displayed in Figure 1. Among statistical packages that perform a SEM analysis, Mplus does not require multivariate normality of the data (Muthen and Muthen, 2007). Therefore, as some dependent variables in this study were categorical, Mplus was appropriate in performing the analysis. The root means square error of approximation (RMSEA), comparative fit index (CFI), and the Tucker-Lewis index (TLI) were utilized to test model fit in Study 1 and Study 2. For Study 2, to determine the effect of childbearing motivation and the mediation of children’s psychological factors on childbearing, the indirect associations were calculated by multiplying the direct associations between each independent latent variable; then, each mediator was multiplied by the estimated association between the mediator and each dependent variable.

## Study 1: Dimensions of Childbearing Motivation

This study investigated the dimensions of parents’ childbearing motivation and tested the invariance of the measurement model. Although several dimensions have been proposed in previous studies, the present measurements were adapted to Chinese culture; thus, two different dimensions were obtained.

### Measures

CFPS respondents (father or mother) were asked to report their childbearing motivations (nine items) on a five-point Likert scale (1 = “Totally agree,” 2 = “Agree,” 3 = “Neutral,” 4 = “Disagree,” 5 = “Totally disagree”). This scale was borrowed from Aycicegi-Dinn and Kagitcibasi’s (2010) research and adapted to the Chinese context. To determine the dimensionality of childbearing motivation, an EFA was conducted. A geomin (oblique) factor rotation was used to increase interpretability of the factors while also enabling the correlation among factors to be determined. Hereafter, a CFA tested the obtained factor structure conjectures and an additional measurement model invariance test was conducted.

### Results

Examination of the eigenvalues suggested a possible two-factor model (eigenvalues greater than one). Model fit was tested utilizing the RMSEA, CFI, and TLI. In terms of the criterion values of these three indexes, a good model has an RMSEA between 0.02 and 0.08, as well as CFI and TLI values close to 1. Specifically, CFI and TLI values above 0.90 suggest an acceptable fit, and those above 0.95 suggest a good model.

The two-factor model demonstrating relatively good fit statistics was selected (RMSEA = 0.108, CFI = 0.963, and TLI = 0.929). Factor loadings and items comprising the factors are presented in Table 2 and shows that factor one contains items 1–3 and factor two contains items 4–9. Based on this factor structure, a CFA was conducted and showed a good model fit (RMSEA = 0.102, CFI = 0.955, TLI = 0.938). Further, there was a significant correlation (*r* = 0.455, *p* < 0.001) between these two factors. Based on the content of the items comprising the four factors, the factors were described as utilitarian motivation (i.e., childbearing motivation focusing on utilitarian benefits of raising a child) and psychological motivation (i.e., childbearing motivation focusing on emotional or psychological benefits of raising a child). This two-factor measurement model was similar to the model developed by Nauck and Klaus (2007).

**Table 2:** Factor loadings for the nine items of childbearing motivation in Study 1.

Item	Factor 1 Utilitarian motivation	Factor 2 Psychological motivation
Raising children is to get support when you are old.	**0.673**	0.000
Raising children is to proliferate your family.	**0.732**	−0.024
Raising children is to obtain financial support from children.	**0.715**	0.011
Raising children is to enjoy the process of raising up children.	−0.023	**0.830**
Raising children is to enjoy the happiness of being accompanied by children.	−0.049	**0.893**
Raising children is to feel the pleasure of having a baby.	0.102	**0.813**
Raising children is to make the family more important in your life.	0.352	**0.584**
Raising children is to increase your sense of responsibility.	0.296	**0.624**
Raising children is to strengthen kinship relations.	0.345	**0.486**
Cronbach reliability α.	0.66	0.83

We used 0.40 as a criterion for inclusion onto a factor. Factor loadings greater than 0.40 are bolded.

To confirm that this measurement model was invariant across the female and male groups, a series of multigroup CFAs were conducted. In the simplest model (the configural model), the same factor structure was required across groups; however, loadings, intercepts, and errors could vary. After this model was estimated, two more restrictive models (weak invariance and strong invariance) required invariance of factor loadings, or additional indicator means, to be estimated. The value of the chi-square test of model fit, degree of freedom, and scaling correction factor for MLR is summarized in Table 3. Based on the Satorra-Bentler scaled chi-square test, the measurement model of childbearing motivation was invariant across groups (*p* configural vs. weak = 0.642, *p* configural vs. strong = 0.524, and *p* weak vs. strong = 0.253). Therefore, the two-factor model of childbearing motivation was invariant across the female and male groups.

**Table 3:** Fit statistics of configural, weak and strong invariance models in Study 1.

	Model fit value	Degree of freedom	Scaling correction factor for MLR
Configural invariance	211.924	52	2.699
Weak invariance	216.523	59	2.7071
Strong invariance	232.309	66	2.5825

## Study 2: the Effect of Parents’ Childbearing Motivation on Children’s Development and the Mediating Role of Children’s Psychological Factors

This study explored the effect of parents’ childbearing motivations on children’s development (academic performance and well-being) and tested the mediating role of children’s psychological factors.

### Measures

#### Children’s Academic Performance

CFPS respondents were asked to report on their children’s general performance in Chinese and Math class last semester. In China, for children aged between 10 and 15 years, Chinese and Math are their major subjects and performance in these two subjects is an important factor in deciding whether they can attend a good university. Academic performance was divided into four levels (1 = “Excellent,” 2 = “Good,” 3 = “Average,” and 4 = “Poor”) with a higher score indicating better performance. Within the study sample, for the subject of Chinese, 24.1% of children reported having “excellent” performance, 32.3% “good” performance, 32.7% “average” performance, and 10.8% “poor” performance. In terms of Math, 26.1% of children reported having “excellent” performance, 28.6% “good” performance, 28.6% “average” performance, and 16.7% “poor” performance.

#### Children’s Well-Being

CFPS respondents were asked to report a self-perceived value of the degree of happiness on an 11-point scale, with a higher score indicating higher psychological well-being. The measurement of well-being was simple because it only had one question; however, there were no other items or questions measuring this concept.

#### Children’s Self-Esteem

CFPS respondents were asked to report self-esteem on a five-point Likert scale (1 = “Totally disagree,” 2 = “Disagree,” 3 = “Agree,” and 4 = “Totally agree”). The scale was adapted from the well-known Rosenberg self-esteem scale (Rosenberg, 1965; see Table 4). Items 3, 5, 9, 10, 11, and 13 were reversed scored. To test the reliability of this measurement within the Chinese context, an EFA was conducted. Results showed that items 1, 2, 4, 6, 7, 8, and 12 loaded on one factor and items 3, 5, 9, 10, 11, and 13 loaded on another factor. Thus, the reverse-coded items may have influenced the consistency of measurement. Thus, to ensure reliable self-esteem measurement, only unreversed items (1, 2, 4, 6, 7, 8, and 12) were retained. The corresponding Cronbach’s *α* was 0.69.

**Table 4:** Factor loadings for the 13 items of self-esteem in Study 2.

Item	Factor 1	Factor 2
I feel that I am a person of worth, at least on an equal plane with others.	0.062	**0.535**
I feel that I have a number of good qualities.	−0.032	**0.634**
All in all, I am inclined to feel that I am a failure. (R)	**0.466**	0.262
I am able to do things as well as most other people.	0.017	**0.444**
I feel I do not have much to be proud of. (R)	**0.313**	0.091
I take a positive attitude toward myself.	0.012	**0.555**
On the whole, I am satisfied with myself.	−0.007	**0.585**
I wish I could have more respect for myself.	−0.009	**0.496**
At times I think I am no good at all. (R)	**0.564**	0.071
I cannot solve current problems. (R)	**0.557**	−0.005
Sometimes I feel forced to do things to make a living. (R)	**0.499**	−0.017
I’m in control of whatever happens to me.	0.044	**0.303**
I feel helpless in my daily life. (R)	**0.410**	−0.082

We used 0.30 as a criterion for inclusion onto a factor. Factor loadings greater than 0.30 are bolded.

#### Children’s Expectation of the Highest Education

CFPS respondents were asked to report a minimum level of education that they thought they should attain. There were seven levels of education: primary school, junior high school, senior high school, 2‐ or 3-year college, 4-year college, or a bachelor’s degree, master’s degree, and doctoral degree. A higher score indicated a higher expectation of education of the child.

### Results

**Figure 2:**
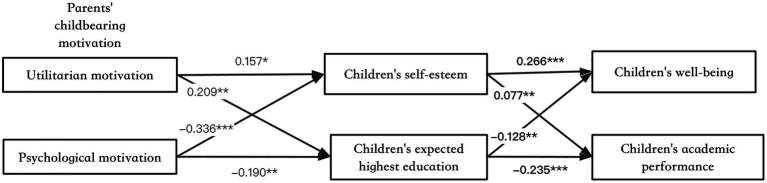
SEM coefficients in the analytical model. Significance level: *p < 0.1, **p < 0.05, and ***p < 0.01.

Table 5 and Figure 2 present standardized SEM coefficients estimated from the model and the model fit indexes, which suggest a sufficient model fit for the overall sample according to the RMSEA, CFI, and TLI values (RMSEA = 0.018; CFI = 0.927 > 0.9; TLI = 0.912 > 0.9). Table 6 presents the standardized direct, indirect, and total associations between each independent latent variable and each dependent variable. Results from the model confirmed the hypotheses that parents’ childbearing motivations have a significant effect on children’s academic performance and well-being. Furthermore, the effect on academic performance was mediated by children expected highest education, while the effect on children’s well-being was mediated by children’s self-esteem. Specifically, children whose parents had a utilitarian motivation regarding childbearing showed poorer academic performance (*b* = −0.271, *p* < 0.001), with this effect being mediated by children’s lower expectation of the highest education (*b* = −0.049, *p* = 0.008). Contrastingly, children whose parents had a psychological motivation regarding childbearing demonstrated better academic performance (*b* = 0.180, *p* = 0.002), with this effect being mediated by children’s higher expectation of the highest education (*b* = 0.045, *p* = 0.014), as well as children’s higher self-esteem (*b* = 0.043, *p* = 0.029). These children also had higher well-being (*b* = −0.116, *p* = 0.025), and this effect was mediated by children’s higher self-esteem (*b* = −0.089, *p* < 0.001). Results of the covariates are intuitive, suggesting that children living in urban areas and those from wealthier families have better academic performance.

**Table 5:** Modeling the associations between parents’ childbearing motivation and children’s academic performance and well-being (standardized structure equation estimates) in Study 2.

	Children’s self-esteem	Children’s expected highest education	Children’s well-being	Children’s academic performance
***Mediators***				
Children’s self-esteem			0.266^∗∗∗^	−0.128^∗∗^
Children’s expected highest education			0.077^∗∗^	−0.235^∗∗∗^
**Parents’ childbearing motivation**				
Utilitarian motivation	0.157^∗^	0.209^∗∗^	0.035	−0.202^∗∗∗^
Psychological motivation	−0.336^∗∗∗^	−0.190^∗∗^	0.767	0.092
***Covariates***				
Living environment (rural vs. urban)	0.024	0.402^∗∗∗^	0.124^∗∗^	−0.165^∗∗∗^
Family income	0.017^∗∗^	0.018^∗∗^	0.003	−0.012^∗∗∗^
**Model statistics**				
Chi-square	540.672^∗∗∗^			
RMSEA	0.018			
CFI	0.927			
TLI	0.912			

∗ *p < 0.1*;

∗∗ *p < 0.05*;

∗∗∗ *p < 0.01*.

**Table 6:** Standardized direct, indirect, and total associations based on the whole study sample in Study 2.

	Children’s academic performance	Children’s well-being
Direct associations with M1	−0.202	0.035
Indirect associations with M1 through SE	−0.020	0.042^∗^
Indirect associations with M1 through EHE	−0.049^∗∗∗^	0.012^∗^
Total indirect associations with M1	−0.069^∗∗∗^	0.053^∗∗^
Total associations with M1	−0.271^∗∗∗^	0.088
Direct associations with M2	0.092	−0.016
Indirect associations with M2 through SE	0.043^∗∗^	−0.089^∗∗∗^
Indirect associations with M2 through EHE	0.045^∗∗^	−0.011^∗^
Total indirect associations with M2	0.088^∗∗∗^	−0.100^∗∗∗^
Total associations with M2	0.180^∗∗∗^	−0.116^∗∗^

M1: Utilitarian motivation (the childbearing motivation focusing on expected utilitarian benefits); M2: Psychological motivation (the childbearing motivation focusing on emotional benefits); EHE: children’s expectation of the highest education and SE: children’s self-esteem.

∗ *p < 0.1*;

∗∗ *p < 0.05*;

∗∗∗ *p < 0.01*.

In comparing the effects of parents’ childbearing motivations in the female and male group, a multigroup analysis was conducted; the results are summarized in Table 7. These results indicate that girls’ academic performance was more likely to be affected by parents’ childbearing motivations, and this effect showed the same pattern as the overall pattern. In terms of well-being, both girls and boys were affected by parents’ childbearing motivations.

**Table 7:** Standardized direct, indirect, and total associations across female group and male group in Study 2.

	Girls’ academic performance	Boys’ academic performance	Girls’ well-being	Boys’ well-being
Direct associations with M1	−0.292^∗∗∗^	−0.132	−0.048	0.100
Indirect associations with M1 through SE	−0.030	−0.011	0.041	0.037
Indirect associations with M1 through EHE	−0.074^∗∗∗^	−0.003	0.028	0.001
Total indirect associations with M1	−0.104^∗∗∗^	−0.013	0.069	0.038
Total associations with M1	−0.396^∗∗∗^	−0.145^∗^	0.021	0.137
Direct associations with M2	0.068	0.132	0.093	−0.131
Indirect associations with M2 through SE	0.069^∗∗∗^	0.022	−0.093^∗∗∗^	−0.076^∗∗^
Indirect associations with M2 through EHE	0.053^∗∗∗^	0.018	−0.020	−0.004
Total indirect associations with M2	0.122^∗∗∗^	0.040	−0.113^∗∗∗^	−0.081^∗∗^
Total associations with M2	0.191^∗∗^	0.173^∗∗^	−0.02	−0.212^∗∗∗^

∗ *p < 0.1*;

∗∗ *p < 0.05*;

∗∗∗ *p < 0.01*.

## Discussion

This study examined the associations between parents’ childbearing motivation and children’s development (academic performance and well-being) with empirical data from 2012 CFPS. Using EFA, this study identified two dimensions of parents’ childbearing motivation, utilitarian and psychological motivation, and tested the invariance of the measurement model across gender. Using SEM, this study tested both direct and indirect relationships between the two different childbearing motivations and children’s development. Results showed that parents’ childbearing motivation focusing on expected utilitarian benefits (utilitarian motivation) decreased children’s expectation of the highest education; thus, worsening children’s academic performance. Alternatively, parents’ childbearing motivation focusing on expected emotional/psychological benefits (psychological motivation) increased children’s expectation of the highest education; thus, improving children’s academic performance. In addition, psychological motivation increased children’s self-esteem; thus, improving children’s well-being.

In this study, a large sample of children in China was analyzed, and the results confirmed the effect of parents’ positive childbearing motivations on children’s academic performance and well-being. It is worth noting that the two dimensions of childbearing motivations of this study identified belong to “positive childbearing motivations” (Guedes et al., 2015), yet these two positive motivations appeared to have had opposite effects on children’s performance and well-being. Compared to prior hypotheses, two main differences were found: (1) parents’ utilitarian motivation did not have a significant effect on children’s well-being and (2) the effect of parents’ psychological motivation on children’s academic performance was mediated by children’s self-esteem irrespective of their expectation of the highest education. The first difference may be because the effect of parents’ utilitarian motivation on children’s academic performance dominates the effect on children’s well-being. That is, there were two dependent variables in the SEM model, so when one dependent variable was investigated, another was controlled for. An independent analysis of children’s well-being showed that children whose parents have utilitarian motivation had significantly lower well-being. The second difference is not surprising as previous literature has confirmed that children with higher self-esteem have better academic performance (Lane et al., 2004; Trautwein et al., 2006). Moreover, the present findings augment existing knowledge regarding the associations between parents’ psychological states or interactions with children on children’s performance or well-being. Previous studies have shown that children’s well-being is affected by parents’ mental health (Mensah and Kiernan, 2010), parenting involvement (Bastaits et al., 2014), high levels of support and control (Baumrind, 2013), and so on. Similarly, this study found that the motivations of bearing a child played an important role in children’s academic performance and well-being.

Additionally, the possible mechanisms behind these results were explored. In the multigroup analysis, a gender difference of the effect was demonstrated, suggesting that girls’ academic performance was more significantly affected by parents’ childbearing motivation. The possible reason for the difference in academic performance is that in China, society has higher expectations on males compared to females due to common beliefs that males should take the main responsibility of supporting a family. Therefore, boys’ academic performance may be more affected by the social norm instead of parent’s expectations.

Findings from this study have several implications for government policies and interventions around family planning and fertility counseling. Expecting children to become better economic providers for their parents in old age is a long-held traditional opinion, especially in underdeveloped areas. This is because when parents retire, governments or the society cannot provide sufficient resources to support them; thus, they can only rely on their children for this support. However, to improve the well-being of the new generation, governments or local organizations could provide mental health services to help parents focus more on the psychological or physical impacts these expectations have on children.

### Limitations and Future Research

Although this study presented the first empirical evidence that parents’ childbearing motivation influences children’s academic performance and well-being, and in turn, children’s expectation of the highest education and self-esteem could explain the effect, several limitations should be considered when interpreting the results.

First, the measure of childbearing motivation in this paper was incomplete. Hoffman and Hoffman (1973) firstly listed nine dimensions of childbearing motivation such as social identity (i.e., the expansion of the self). Guedes et al. (2015) classified various motivations into four types: emotional or psychological, social or normative, economic or utilitarian, and biological or physical motivation. The two dimensions of childbearing motivation used in this study were subsets of the motivations investigated in previous research. Thus, this study did not provide a full assessment of how different childbearing motivations influence children’s development. Therefore, future research should examine the various effects of different childbearing motivations and their corresponding mechanisms in more depth to expand the present findings.

Second, the study design was cross-sectional in nature and did not offer strong casual inferences. Although parents’ childbearing motivation seems to be formed before children are born, this motivation may also change during interactions with their children. For example, a child with a good personality and behavior makes his or her parents feel greater joy from parenthood. Therefore, future studies should implement a longitudinal study or behavioral experiment to investigate how parents’ childbearing motivation shapes children’s development over time.

Finally, additional mechanisms must be considered. To illustrate, in this study, children’s expectation of highest education and self-esteem were used to explain the effect of parents’ childbearing motivation on children’s academic performance and well-being, respectively. However, children’s development is affected by various psychological factors; thus, the mechanisms may be different for girls and boys. For example, psychological motivation had a positive effect on boys’ academic performance; however, the effect was not mediated by their expectation of the highest education or self-esteem. A topic for future study is thus to explore the different mechanisms of parents’ childbearing motivations and their effect for girls and boys. Overall, we hope the current study provides a foundation for future investigations into how parents’ childbearing motivation influences the development of the next generation.

## Conclusion

These findings have provided important insights into how positive childbearing motivations influence children’s development. Furthermore, the influence of self-esteem and expectation of the highest education were revealed as important mediators in this relationship. Therefore, these findings can be utilized to increase awareness of the effect of childbearing motivations on children’s development; thus, ensuring better development for the next generation.

## Data Availability Statement

The raw data supporting the conclusions of this article will be made available by the authors, without undue reservation.

## Ethics Statement

The studies involving human participants were reviewed and approved by China National Knowledge Infrastructure (CNKI) Ethics Committee. Written informed consent to participate in this study was provided by the participants’ legal guardian/next of kin.

## Author Contributions

All authors listed have made a substantial, direct and intellectual contribution to the work, and approved it for publication.

### Conflict of Interest

The authors declare that the research was conducted in the absence of any commercial or financial relationships that could be construed as a potential conflict of interest.
